# Regulation of Structure-Specific Endonucleases in Replication Stress

**DOI:** 10.3390/genes9120634

**Published:** 2018-12-14

**Authors:** Seong Min Kim, Susan L. Forsburg

**Affiliations:** Program in Molecular & Computational Biology, University of Southern California, Los Angeles, CA 90089, USA; seongmk1@gmail.com

**Keywords:** replication stress, structure-specific endonuclease, Mus81, XPF

## Abstract

Replication stress results in various forms of aberrant replication intermediates that need to be resolved for faithful chromosome segregation. Structure-specific endonucleases (SSEs) recognize DNA secondary structures rather than primary sequences and play key roles during DNA repair and replication stress. Holliday junction resolvase MUS81 (methyl methane sulfonate (MMS), and UV-sensitive protein 81) and XPF (xeroderma pigmentosum group F-complementing protein) are a subset of SSEs that resolve aberrant replication structures. To ensure genome stability and prevent unnecessary DNA breakage, these SSEs are tightly regulated by the cell cycle and replication checkpoints. We discuss the regulatory network that control activities of MUS81 and XPF and briefly mention other SSEs involved in the resolution of replication intermediates.

## 1. Introduction

The DNA replication fork is sensitive to a variety of intrinsic and extrinsic stresses (reviewed in [1,2]). Endogenous blocks include collisions with transcription apparatus, natural pausing sites, and unusual DNA structures or sequences (reviewed in [3]). Highly repetitive DNA sequences (e.g., ribosomal DNA, telomeres) or common fragile sites (CFS) are also more prone to replication stress (reviewed in [4,5]). External agents that disrupt replication include depletion of deoxyribonucleotide triphosphate (dNTP) by hydroxyurea (HU) and DNA lesions caused by ultraviolet (UV) radiation, alkylating agents such as methyl methane sulfonate (MMS), or the topoisomerase inhibitor camptothecin (CPT) (reviewed in [1]).

Replication stress can result in accumulation of single stranded DNA, chromosome breaks, and rearrangements, which are deleterious to the cell (reviewed in [1,2]). Additionally, it may generate aberrant intermediates including DNA secondary structures, DNA lesions, and protein-DNA complexes (reviewed in [4]). Not surprisingly, increased replication stress is now recognized as a contributor to oncogenesis (e.g., reviewed in [6,7]).

**Figure 1:**
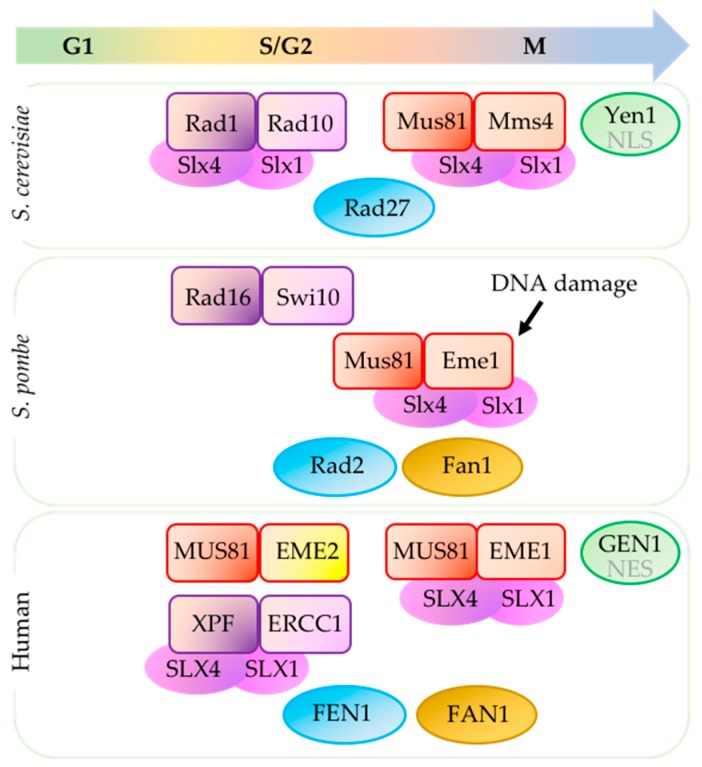
Structure-specific endonucleases (SSEs) in different phases of the cell cycle. Mus81 (methyl methane sulfonate (MMS) and UV-sensitive protein 81) activity in *Saccharomyces cerevisiae* and human cells is stimulated during G2-M transition (reviewed in [8]). In *Schizosaccharomyces pombe,* Mus81 is activated by DNA damage. Xeroderma pigmentosum group F complementing protein (XPF)-excision repair cross-complementing group 1 (ERCC1) (orthologs Rad1-Rad10*^S.c.^* and Rad16-Swi10*^S.p.^*) is important for various DNA repair pathways and cleaves replication intermediates during S and G2 phases [10]. Scaffold protein SLX4 with associating partner SLX1 interacts with MUS81-EME1 (essential meiotic endonuclease 1) and XPF-ERCC1 in human cells (reviewed in [11]) [12,13,14,15] and their orthologs in in *S. cerevisiae* (reviewed in [16]) [17,18,19,20]. In contrast, Slx4 does not affect Rad16-Swi10 in *S. pombe* [21]. Activity of Yen1*^S.c.^* is prevented until anaphase by restricting its nuclear entry due to phosphorylation of nuclear localization signal (NLS) [22,23,24]. Due to nuclear export signal (NES), GEN1 in human cells is able to access chromosomes only after nuclear membrane breakdown during mitosis [25]. *S. pombe* do not have Yen1 ortholog (reviewed in [26]). FEN1 (flap endonuclease 1) (orthologs Rad27*^S.c.^* and Rad2*^S.p.^*) and FAN1 (Fanconi-associated nuclease I) (missing in *S. cerevisiae*) contribute to processing replication intermediates but cell cycle-dependent regulation of these SSEs are not well characterized (reviewed in [8]). Mms4 (methyl methane sulfonate sensitivity protein 4).

A subset of structure-specific endonucleases (SSEs) that recognize specific DNA structures rather than DNA sequences, plays a crucial role in processing these aberrant structures to ensure replication fork stability and progression (reviewed in [8]). These SSEs are essential to maintaining genome stability, coordinating with the cell cycle to ensure that cells do not enter mitosis with structures that would promote improper chromosome segregation and breakage (reviewed in [9]). In this review we describe SSEs involved in processing DNA replication intermediates directly or indirectly regulated by the replication checkpoint (Figure 1). We pay particular attention to two conserved, related SSEs: Mus81 (MMS and UV-sensitive protein 81) and XPF (xeroderma pigmentosum group F-complementing protein).

## 2. Mus81-Essential Meiotic Endonuclease 1 (*Schizosaccharomyces pombe*)/Mus81-Mms4 (*Saccharomyces cerevisiae*)/MUS81-EME1/2 (Human)

### 2.1. Mus81 Processes Replication and Recombination Intermediates

The Mus81 protein was identified for its role in processing complex branched DNA structures, including Holliday junctions, that form after complementary strand exchange between homologous sequences (reviewed in [27,28,29,30,31,32]). Mus81 can resolve synthetic Holliday junction structures in vitro [31,32] and has a high affinity for branched duplex DNA and replication fork substrates [33]. Consistent with this, loss of *mus81* leads to severed meiotic defects, resulting in abnormal chromosomal segregation defects in yeasts [31,34,35]. In fission yeast (*Schizosaccharomyces pombe*), it is essential to complete sister chromatid exchange at the mating locus [31,36].

Mus81-dependent resolution of entangled sister chromatids is essential for survival of cells that depend on homology-directed repair of collapsed replication forks [36,37]. In human cells, MUS81 is similarly needed for replication fork restart after replication stress inducing agents [38,39,40,41]. MUS81-deficient cells have decreased viability upon low-dose exposure to these replication inhibitors [41]. Importantly, fork restart in BRCA2 (breast cancer–associated protein 2)-deficient cells requires MUS81-dependent cleavage of partially resected, regressed forks [42]. In addition to resolving replication intermediates, compensatory DNA synthesis during mitosis and cleavage of mitotic interlinks to allow chromosomal segregation also require MUS81 [43]. These and many more studies demonstrate Mus81 plays a critical role to resolve replication and recombination intermediates and ensure proper chromosome segregation during cell division.

But Mus81 is a double-edged sword. Unregulated activity can have deleterious effects. Mus81 causes replication stress-induced double stranded breaks (DSB) in mammalian cells [38] and promotes deletion mutations in *polα* mutant fission yeast [44]. When an active replication fork converges on a collapsed fork, replication termination is prone to Mus81-dependent deletions between repetitive DNA sequences in fission yeast [45]. In human cells, oncogene-induced chromosomal breakage involves MUS81 activity [46]. These findings suggest that tight regulation of Mus81 is necessary to repair replication-associated DNA structures without inducing unnecessary DNA cleavage.

### 2.2. Regulation of Mus81 by Cell Cycle Kinases

**Figure 2:**
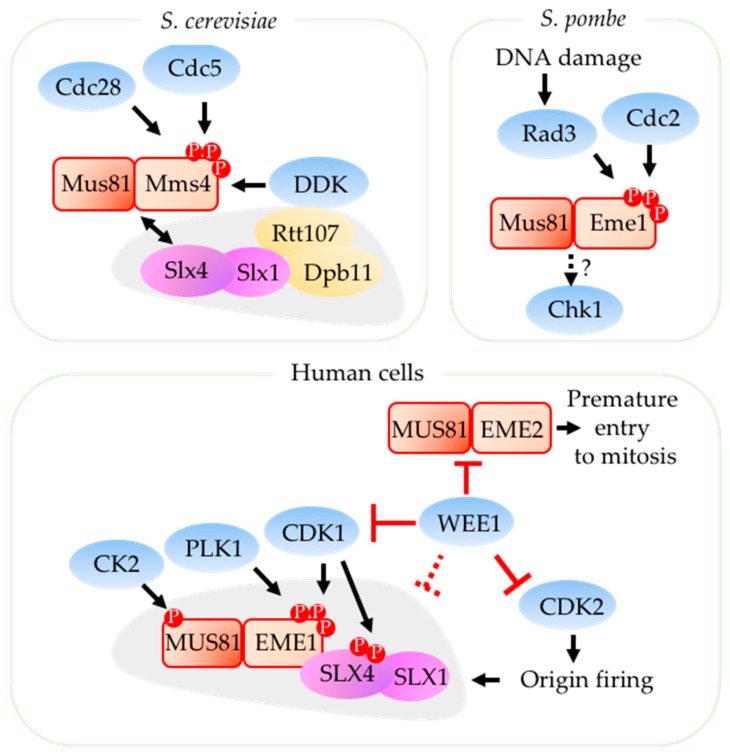
Mus81 regulation by cell cycle kinases. In *S. cerevisiae*, Mus81-Mms4 is phosphorylated by Cdc28*^S.c.^* (CDK1 ortholog) and Cdc5*^S.c.^* (PLK ortholog) kinases at the G2/M transition [48,49,50]. Scaffold protein Rtt107*^S.c.^* (PTIP ortholog) associates with Dpb11*^S.c.^* (TOPBP1 ortholog) and interacts with DDK which also phosphorylates Mms4*^S.c.^* [52]. Rtt107-Dpb11-Slx4*^S.c.^* complex associates with Mus81-Mms4*^S.c.^* behind replication forks. In *S. pombe*, Mus81-Eme1*^S.p.^* is phosphorylated by Cdc2*^S.p.^* (CDK1 ortholog) which primes Eme1*^S.p.^* for phosphorylation by Rad3*^S.p.^* (ATR ortholog) upon DNA damage [53]. Mus81-Eme1*^S.p.^* may be contributing to Chk1 activation in fission yeast as Mus81-deleted cells with replication defect are able to bypass Chk1 checkpoint [54]. In human cells, MUS81-EME1 activity peak during M phase after EME1 is phosphorylated by CDK1, PLK1 [12,58,59]. SLX4 phosphorylation by CDK1 and MUS81 phosphorylation by CK2 also promotes MUS81-EME1 activity [60]. During S-phase, WEE1 downregulates MUS81-EME1 activity by inhibiting CDK1 and thereby limiting EME1 and SLX4 phosphorylation (reviewed in [56]). WEE1 inhibition of CDK2 reduces origin firing and subsequently the replication intermediate substrates of MUS81. WEE1 may also inhibit MUS81 directly [61]. Residual MUS81 activity during S-phase comes from MUS81 that forms complex with EME2 which can promote premature entry to mitosis upon WEE1 inhibition [62].

A key component of that regulation is cell cycle- and checkpoint-dependent regulation of Mus81. These restrict its activity to later in the cell cycle in unstressed cells. The Mus81 enzyme forms a complex with Eme1 (essential meiotic endonuclease 1) which creates a stable interaction with a DNA substrate for the complex [47]. Phosphorylation of Eme1 by various cell cycle kinases provide one mechanism to regulate Mus81 activity. In budding yeast (*Saccharomyces cerevisiae*), Mus81 forms a complex with the Eme1 ortholog Mms4 (methyl methane sulfonate sensitivity protein 4) [8]. Mus81-Mms4*^S.c.^* is activated in a cell cycle-dependent manner and depends on phosphorylation of Mms4*^S.c.^* by the cell cycle kinases Cdc28*^S.c.^* (CDK1 in human) and Cdc5*^S.c.^* (PLK1 in human) at the G2/M transition (Figure 2) [48,49,50]. This restricts Mus81-Mms4*^S.c.^* activity during S-phase to prevent unnecessary cleavage of DNA substrates while DNA replication is occurring [48,51]. Via the scaffold protein Rtt107*^S.c.^*, Cdc7-Dbf4*^S.c.^* (Dbf4-dependent kinase, DDK) interacts with and phosphorylates Mus81-Mms4, which is required for Mus81 activation during mitosis [52].

In fission yeast, which spends of most of its lifetime in G2 phase, Mus81-Eme1*^S.p.^* activity is upregulated in response to DNA damage [8]. Cdc2*^S.p.^* (CDK1 in human) phosphorylation of Eme1*^S.p.^* primes it for phosphorylation and activation by the DNA damage sensor and checkpoint activator Rad3*^S.p.^* (ATR in human) (Figure 2) [53]. Mus81-Eme1 cleavage of replication intermediates in in turn may have a role in activation or propagation of checkpoint pathways. Deletion of Mus81*^S.p.^* in replication stress-induced, temperature-sensitive Mcm4 helicase mutant (*mcm4-ts*) results in failure to maintain the DNA damage checkpoint and in subsequent abnormal chromosomal segregation [54].

In human cells, MUS81 is up-regulated at the onset of mitosis and has two partners, EME1 and EME2 [8,55]. Approximately 80% of MUS81 is associated with EME1 while the remaining 20% is associated with EME2 (reviewed in [56]). It is not obvious whether EME1 or EME2 is responsible for S phase-specific functions of MUS81 [55,57]. Interestingly, MUS81-EME1 activity is needed for maintaining replication fork speed [57] while MUS81-EME2 activity promotes replication fork restart and chromosomal stability [55].

MUS81-EME1 activity in human cells peaks during M phase after hyperphosphorylation of EME1 by cell cycle kinases CDK1 and PLK1 (Figure 2) [12,58,59]. Uninhibited CDK1 activity results in chromosomal fragmentation following premature activation of MUS81 [63], further linking CDK to MUS81 activity. PLK1 promotes DNA repair protein BRCA1 recruitment to facilitate MUS81-mediated fork cleavage coupled with a break-induced replication [64]. Moreover, PLK1 interaction with BRCA1 and CDK1 activation of RECQ5 DNA helicase promotes MUS81-EME1 recruitment to CFS [65]. A recent study showed that the pleiotropic serine/threonine kinase CK2 kinase is able to phosphorylate MUS81 in late-G2/mitosis and upon mild replication stress to promote its association with EME1 and scaffold protein SLX4, another stimulator of MUS81 activity [60]. These findings show that cell cycle-dependent kinases not only play a crucial role in restricting Mus81 activity to appropriate timing of the cell cycle but also contribute to Mus81-dependent DNA repair.

**Figure 3:**
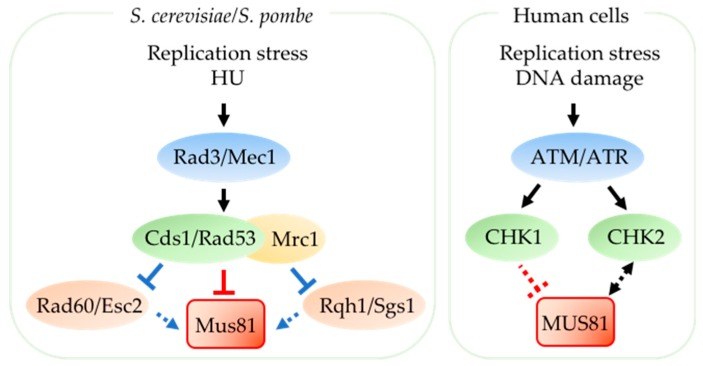
Mus81 regulation by replication checkpoint. In yeast, replication stress induces Rad3*^S.p.^*/Mec1*^S.c.^* (ATR in human) activation of Cds1*^S.p.^*/Rad53*^S.c.^* by promoting its association with Mrc1^S.*p./S.c.*^ (CLASPIN in human) (reviewed in [80]). Upon acute and severe replication stress such as hydroxyurea treatment, Cds1*^S.p.^* limits Mus81*^S.p.^* activity (indicated by solid red line) [44]. Cds1*^S.p.^* inhibits Rad60*^S.p.^* activity (indicated by blue line) by promoting delocalization from the nucleus [82,83]. Mrc1*^S.p./S.c.^* protein level regulates recruitment of Rqh1*^S.p.^* homolog Sgs1*^S.c.^* to chromatin (indicated by blue arrow) [74]. Both Rad60*^S.p.^*/Esc2*^S.c.^* and Rqh1*^S.p.^*/Sgs1*^S.c.^* contribute to Mus81 activity (indicated by dashed blue arrow) [75,76]. In human cells, DNA damage checkpoint CHK1 and Cds1-homolog CHK2 is activated downstream of ATM/ATR kinases (reviewed in [79,80]) [77]. It is unclear whether MUS81 is directly regulated by these checkpoint kinases in human cells. However, there is evidence that CHK2 upregulates MUS81 protein levels and MUS81 in turn contributes to CHK2 activation upon DNA damage (indicated by dashed double-headed arrow) [84]. Deleterious MUS81-dependent processing of replication intermediates following CHK1 inhibition suggests that CHK1 downregulates MUS81 activity (indicated by dashed red line) [68,69,70].

Other regulators down-regulate S-phase activity of MUS81. WEE1, a well-known inhibitor of CDKs, suppresses MUS81 activity during S-phase by: (1) Potentially phosphorylating MUS81, (2) by inhibiting CDK2 and thereby limiting origin firing and replication stress, and (3) by restraining CDK1 that phosphorylates and activates EME1 and scaffold protein SLX4 (Figure 2) (reviewed in [56]). In the absence of WEE1, MUS81-EME1 activity results in unnecessary replication fork cleavage, leading to accumulation of DNA damage [61,66]. Deletion of MUS81 in the absence of WEE1 reduces DSB [61] but does not prevent activation of ATR and CHK1 [67], suggesting that MUS81 activity is downstream of replication fork stalling and S-phase checkpoint. This is also evidenced by the detrimental MUS81-dependent processing of replication intermediates following CHK1 inhibition [68,69,70]. Although the mechanistic details are unknown, these findings indicate that CHK1 down-regulates MUS81 in human cells (Figure 3).

Unlike CDK1-and PLK1-regulated control of MUS81-EME1 activity, the control of MUS81-EME2 activity is not well-established despite the evidence that MUS81-EME2 is responsible for the DNA damage during premature entry to mitosis upon WEE1 inhibition [62]. Because deletion of MUS81 or EME2 delays premature entry into mitosis induced by WEE1 inhibition, this suggests that regulating MUS81-EME2 activity may be the mechanism by which WEE1 prevents premature mitotic entry (Figure 2) [8,62].

### 2.3. Mus81 is Regulated by the Replication Checkpoint during Replication Stress

During replication stress, Mus81 plays a crucial role in processing abnormal replication intermediates. It is recruited to sites of replication blockage to resolve replication intermediates and inhibits anaphase bridge formation, preventing chromosome mis-segregation and transmission of damaged DNA to daughter cells (reviewed in [71,72,73]). Loss of Mus81 attenuates recovery of stalled replication forks and makes cells hypersensitive to DNA damaging agents that obstruct replication fork progression [29,38,39,74,75,76,77]. Paradoxically, although Mus81 is required to resolve aberrant replication intermediates, it can also create DNA breaks that threaten genomic stability. This is why Mus81 regulation during replication stress is crucial. Upon replication disturbance, the replication checkpoint pathway is activated to resolve replication hindrances and to delay mitosis until the replication stress is relieved (reviewed in [27,78]).

Cds1*^S.p.^* is the fission yeast replication checkpoint effector (Figure 3). In budding yeast, the Cds1*^S.p.^* homolog Rad53*^S.c.^* is the effector of both the DNA damage checkpoint and the replication checkpoint. Fission yeast Cds1*^S.p.^* acts downstream of DNA-dependent protein kinase-like family Rad3*^S.p.^* (Mec1*^S.c.^*/ATR in human) (reviewed in [79]). Upon replication stress, a conserved mediator protein Mrc1*^S.p.^* (CLASPIN in human) is phosphorylated by Rad3*^S.p.^* which then recruits Cds1*^S.p.^* to stalled replication forks to be activated (reviewed in [80,81]).

In fission yeast, Cds1*^S.p.^* is necessary to prevent accumulation of aberrant replication intermediates, indicating that Cds1*^S.p.^* regulates resolution of damaging DNA structures at replication forks (reviewed in [85]) [86,87,88,89,90,91]. Mus81*^S.p.^* is a key target downstream of Cds1*^S.p.^* [29,31,32,44]. This S-phase checkpoint kinase tightly regulates Mus81*^S.p.^* to prevent uncontrolled nuclease activity during DNA replication. Mus81*^S.p.^* associates with 2–5% of the Cds1*^S.p.^* protein through a forkhead-associated (FHA) domain on Cds1*^S.p.^* and is phosphorylated in Cds1*^S.p.^*-dependent manner upon replication stress [29,92]. Acute HU treatment results in phosphorylated Mus81*^S.p.^* dissociating from the chromatin to prevent extensive cleavage of replication intermediates [44]. Loss of Cds1-Mus81*^S.p.^* interaction via mutations in the FHA domain of Cds1*^S.p.^* or FHA-binding site on Mus81*^S.p.^* abolishes HU-induced Mus81*^S.p.^* phosphorylation, so that Mus81*^S.p.^* remains associated with chromatin with deleterious effects [44]. On the other hand, chronic low dose HU-treatment reduces Cds1*^S.p.^*-mediated inhibition of Mus81*^S.p.^* activity and this allows processing of DNA secondary structures that form during extended replication block [44]. Mus81*^S.p.^* is also required for Cds1*^S.p.^*-mediated slowing of replication upon MMS treatment [93]. 

In addition to phosphorylating Mus81*^S.p.^*, Cds1*^S.p.^* may also indirectly modulate Mus81*^S.p.^* activity by regulating proteins that function closely with Mus81*^S.p.^* to respond to damage during replication (Figure 3). For instance, the DNA repair protein Rad60*^S.p.^* and RecQ family DNA helicase Rqh1*^S.p.^* function coordinately with Mus81*^S.p^* in recombinational repair (reviewed in [78]). Cds1*^S.p.^* down-regulates nuclear localization of Rad60*^S.p.^* in replication-arrested cells [82,83] and small ubiquitin-related modifier (SUMO)-like domains of Rad60*^S.p^* homolog Esc2*^S.c.^* in budding yeast are critical for stimulation of Mus81*^S.c.^* [75]. Moreover, Rqh1*^S.p.^* contributes to the formation of Mus81*^S.p.^*-mediated DSB in Cds1*^S.p.^*-deleted cells [76]. In budding yeast, Cds1*^S.p.^*/Rad53*^S.c.^* activator Mrc1*^S.p./S.c.^* protein level regulates recruitment of Rqh1*^S.p.^* homolog Sgs1*^S.c.^* to chromatin [74]. Cds1*^S.p.^*/Rad53*^S.c.^* regulation of Rad60*^S.p.^*/Esc2*^S.c.^*, Rqh1*^S.p^^.^*/Sgs1*^S.c.^*, and possibly other proteins involved in resolving replication fork stress may be an important coordinator of Mus81*^S.p^* activity.

In mammalian cells, DNA damage checkpoint kinase CHK1 and Cds1*^S.p.^* -homolog CHK2 are activated downstream of ATM/ATR kinases in response to certain replication blocks and to DNA damage during S-phase (reviewed in [79]) [77]. Although Cds1-Mus81*^S.p.^* interaction is conserved in human cells (CHK2-MUS81), it is unclear if CHK2 directly regulates MUS81 as in fission yeast (reviewed in [8]), although there is evidence that CHK2 up-regulates the protein level of MUS81; MUS81 in turn contributes to activation of CHK2 in Cisplatin-treated breast cancer cells (Figure 3) [84].

### 2.4. Other Regulators of Mus81 Recruitment and Activity

There is growing evidence that there are other regulators of Mus81 activity besides cell cycle and replication checkpoint kinases (Table 1). For example, the N-terminal fragment of DNA repair protein Rad52*^S.c.^* stimulates the endonuclease activity of Mus81-Mms4*^S.c.^* on homologous recombination intermediates in budding yeast [94]. RAD52 also promotes MUS81-mediated break-induced replication repair of collapsed forks and mitotic DNA synthesis in human cells [95,96]. The small ubiquitin-related modifier (SUMO)-like domain of the adaptor protein establishment of silent chromatin 2 (Esc2*^S.c.^*) in budding yeast interacts with and stimulates Mus81-Mms4*^S.c.^* catalytic activity [75]. The replication factor C (RFC) complex and the loading of proliferating cell nuclear antigen (PCNA) also enhances recruitment and activity of Mus81-Mms4*^S.c.^* [97].

Interestingly, the Structural Maintenance of Chromosomes (SMC) complexes are another modulator of Mus81^S.*p/S.c.*^ activity. In yeast, for example, the Smc5-Smc6^S.*p/S.c.*^ complex promotes Mus81^S.*p/S.c.*^-dependent resolution of Holliday junctions [99,100]. The positive genetic interactions between certain mutants affecting methylation of cohesin subunit Psm1*^S.p.^* and Mus81-Eme1*^S.p.^* mutants in fission yeast suggests that methylation of cohesin subunits may be important for Mus81 activity at the stalled replication fork. Alternatively, Mus81 may be required for recruitment of the cohesin to sites of DNA damage [102]. In human cells, depletion of SMC2, which is required for chromosome condensation, or WAPL (Wings apart protein-like), which is required for release of sister-chromatid arm cohesin, results in failure to recruit MUS81 to chromatin [101].

In human cells, post-translational modification of MUS81 other than phosphorylation may be important for its activity during DNA repair. This is evidenced by compromised DNA damage response in cells with SUMOlyation-resistant MUS81 upon arsenic treatment that mimic metal carcinogenesis [105]. Epigenetic modifications adjacent to replication forks may also contribute to regulation of MUS81 recruitment and activity. For instance, EZH2 (enhancer of zeste homologue 2) that methylates histone H3 on Lys27 (H3K27) at stalled replication forks has been shown to mediate recruitment of MUS81 [106].

Localization of MUS81 is another way its activity is modulated. In human cells, MUS81 accumulates in the nucleolus during S phase, suggesting that it is required to maintain highly repetitive nucleolar DNA (reviewed in [8]). MUS81 relocates from the nucleolus to localized regions of UV damage specifically in S-phase cells [103]. Sub-localization of Mus81*^S.c.^* also occurs in budding yeast. Following DNA damage, Mus81-Mms4*^S.c.^* relocalizes to subnuclear foci and colocalizes with other endonucleases and with Cmr1*^S.c.^*, a protein involved in genome stability maintenance [104]. Subnuclear Mus81-Mms4*^S.c.^* foci persist until the resolution of accumulated DNA intermediates following DNA damage [104].

These findings demonstrate that cells are equipped with multiple means to tightly regulate Mus81 recruitment and activity. Investigating how these various modulators of Mus81 communicate with each other will further elucidate Mus81-dependent genome stability maintenance.

## 3. Rad16-Swi10 (*Schizosaccharomyces pombe*)/Rad1-Rad10 (*Saccharomycescerevisiae*)/Xeroderma Pigmentosum Group F Complementing Protein (XPF)-Excision Repair Cross-Complementing Group 1 (ERCC1) (Human)

An additional SSE plays a related role to Mus81. Xeroderma pigmentosum group F complementing protein (XPF)-excision repair cross-complementing group 1 (ERCC1) heterodimer complex is a 5ˇä-3ˇä structure-specific endonuclease involved in a variety of DNA repair pathways including nucleotide excision repair (NER) and has important roles in interstrand crosslink (ICL) repair and DSB repair (reviewed in [125]). Rad16-Swi10*^S.p.^* is the fission yeast ortholog and Rad1-Rad10*^S.c.^* is the budding yeast ortholog (reviewed in [8]).

With MUS81-EME1, XPF-ERCC1 processes under-replicated DNA and replication intermediates at CFS and prevents anaphase bridges following recruitment by SLX4 [72,73]. However, MUS81 and XPF may differ in the timing of their activity (Figure 1). In human cells, the biological function of MUS8-EME1 is mostly during mitosis although MUS81 activity is present throughout the cell cycle, probably through its association with EME2 (reviewed in [56]). During S- and G2-phase, XPF-ERCC1 along with another endonuclease ARTEMIS, are responsible for replication stress-induced fork cleavage needed to resume DNA replication [10]. Data from fission yeast indicates that Mus81*^S.p.^* and Rad16*^S.p.^*/XPF may direct repair towards different templates, with Mus81*^S.p.^* using the sister chromatid and Rad16*^S.p.^*/XPF using ectopic sequences [36].

Despite minimal overlap in substrate specificity in vitro [126], evidence suggests that XPF and MUS81 provide overlapping activity. Mammalian XPF is not required for viability [127,128], possibly due to overlap with MUS81 or other SSEs [129,130,131]. XPF becomes essential in chicken DT40 cells if MUS81 is missing [132]. In fission yeast, a double mutant lacking both Rad16*^S.p.^*/XPF and Mus81*^S.p.^* is inviable [21]. Consistent with this, XPF-ERCC1 partially compensates for MUS81 loss during mild replication stress in mammalian cells [41]. MUS81-EME1 also rescues the viability of XPF-deleted cells [132].

Association with different recruiting partners and stimulating proteins appears to determine in which repair pathway XPF-ERCC1 will function (Table 1). In fission yeast, a recently identified protein Pxd1*^S.p.^* (*pombe* XPF and Dna2) stimulates 3ˇä-endonuclease activity of Rad16-Swi10*^S.p.^* [113]. In budding yeast, Saw1*^S.c.^* (Single-strand annealing weakened protein 1), a structure-specific DNA binding protein, recruits Rad1-Rad10*^S.c.^* to single-strand annealing repair sites [114] while damage recognition protein Rad14*^S.c.^* brings Rad1-Rad10*^S.c.^* to NER [109]. In human cells, ERCC1 cannot enter the nucleus without XPF, demonstrating that XPF-ERCC1 heterodimer formation is critical [133]. In NER, XPF-ERCC1 cleavage of the damaged stand is stimulated by RPA and Rad52 [110,112]. RPA is also required for XPF-ERCC1 endonuclease activity in replication-coupled ICL repair [111]. In human cells, both XPF-ERCC1 and MUS81-EME1 are recruited to the replication fork stalled at ICL by the scaffold protein SLX4 and this depends on ubiquitylation of the FANCD2 (Fanconi anaemia complementation group D2) (reviewed in [98]) [134,135]. Independently of SLX4, the scaffold protein UHRF1 (ubiquitin-like PHD and RING finger domain-containing protein1) is needed to recruit FANCD2 and MUS81-EME1 and XPF-ERCC1 to DNA damage sites [107,108].

## 4. Structure-Specific Endonuclease Subunit Slx4 (*Schizosaccharomyces pombe*)/Slx4 (*Saccharomyces cerevisiae*)/SLX4 (Human)

**Figure 4:**
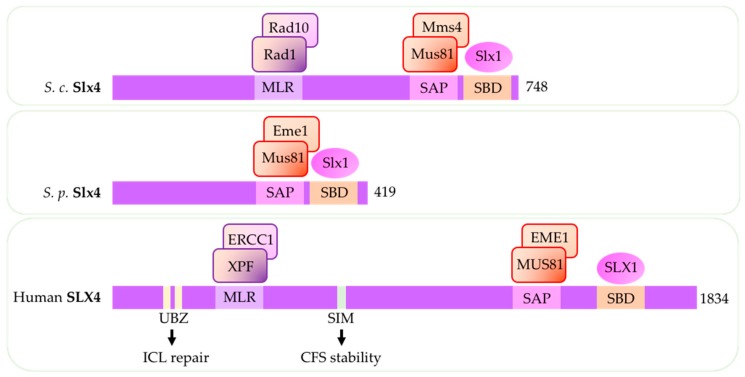
Important domains in SLX4. Across all three species (*S.p.*, *S.c.*, and human), scaffold protein SLX4 have SLX1 binding domain (SBD) and MUS81-EME1 binding region (SAP) [58]. Slx4*^S.c.^* in budding yeast and SLX4 in human cells also have XPF-ERCC1 interacting region MLR. Slx4*^S.p.^* in fission yeast lack MLR. SLX4 in human cells have UBZ (ubiquitin-binding zinc finger domain) and SIM (SUMO-interaction motif )motifs that contributes to its recruitment and activity [135,136]. (SBD: SLX1 binding domain; SAP: SAF-A/B, Acinus and PIAS domain that interacts with MUS81-EME1; MLR: MEI9XPF-interaction-like region that interacts with XPF-ERCC1; ICL: interstrand crosslink; CFS: common fragile site) (*S.c.: S. cerevisiae; S.p.: S. pombe*).

In human cells and in budding yeast, SLX4/Slx4*^S.c.^* forms a complex with its interacting partner SLX1/Slx1*^S.c.^* and, as previously mentioned, serves as a binding platform and catalytic stimulator for both MUS81-EME1/Mus81-Mms4*^S.c.^* and XPF-ERCC1/Rad1-Rad10*^S.c.^* (Figure 4) (reviewed in [11]) [12,13,14,15]).

In budding yeast, upon replication stress, Slx4*^S.c.^* forms a complex with another scaffold protein Rtt107*^S.c.^* (PTIP *^H.s.^*) and DNA replication initiation protein Dpb11*^S.c.^* (TOPBP1*^H.s.^*) and associates with Mus81-Mms4*^S.c.^* behind replication forks after Cdc28*^S.c.^*-and Cdc5*^S.c.^*-mediated phosphorylation of Mms4 *^S.c.^* [16,17,18,19,20]. In Mus81*^S.c.^*-deficient cells, Slx4*^S.c.^* play a critical role supporting replication-coupled ICL repair by Rad1-Rad10*^S.c.^* [137]. In contrast to budding yeast, Slx4*^S.p.^* in fission yeast lacks the XPF-interacting region (Figure 4) and does not appear to affect Rad16*^S.p.^* as Slx4*^S.p.^* deletion has no sensitivity to UV or MMS and no synthetic growth defects with Rad16*^S.p.^* mutation [21].

In human cells, increased association of MUS81-EME1 with the scaffold protein SLX4 contributes to MUS81-dependent processing of DNA secondary structures [12]. SLX4 deletion reduces MUS81-dependent formation of DSBs that occur after WEE1 inhibition [62,67]. SLX4 is phosphorylated by CDK1 in late G2 and M phase and interacts with MUS81-EME1 complex and SLX1, forming stable SLX-MUS complex (reviewed in [49,62]. SLX4 recruitment to chromatin and SLX4-mediated sister chromatid resolution requires TOPBP1 [138]. In addition to recruiting MUS81-EME1 and XPF-ERCC1, nuclease activity of SLX4 is important for processing telomeric structures and oppose aberrant telomere synthesis observed in cancers (reviewed in [139]) [140,141]. SLX4 also suppresses chromatin association with another SSE, GEN1(Yen1), in the absence of MUS81 and prevent DSBs after pathological replication stress [142]. Human SLX4 has ubiquitin-binding zinc finger (UBZ) motif and SUMO-interaction motif (SIMs) (Figure 4) [135,136]. The UBZ motif is required for SLX4 recruitment to sites of replication-dependent ICL repair while the SIMs is required for the function of SLX4 during replication stress and in suppressing CFS instability [143,144].

## 5. Other Structure-Specific Endonuclease in Replication Stress

Although MUS81-EME1 and XPF-ERCC1, along with the scaffold protein SLX4, are the most well-characterized SSEs to be responsible for processing replication intermediates, a few other SSEs have been noted to be important in dealing with replication stress.

### 5.1. Rad2 (*Schizosaccharomyces pombe*)/Rad27 (*Saccharomyces cerevisiae*)/FEN1 (Flap Endonuclease 1) (Human)

Flap endonuclease 1 (FEN1 in human, Rad2*^S.p.^* in fission yeast, Rad27*^S.c.^* in budding yeast) has an important role of removing 5ˇä flaps that form during Okazaki fragment maturation via its interaction with DNA processivity factor PCNA (Table 1) (reviewed in [115]). FEN1 is also involved in processing DNA secondary structures during replication fork impediment, especially in rDNA and telomeres [145,146,147]. This process requires FEN1 to undergo SUMOylation and subsequent interaction with the PCNA-like Rad9-Rad1-Hus1 complex [116,124]. FEN1 and MUS81 associate with each other and collaborate in removing various aberrant DNA structures, including regressed replication fork substrates [117,118,119]. FEN1 removes the 5ˇä-flap after MUS81 processes DNA junction structures (reviewed in [78]) [86]. This process requires FEN1 to be stimulated by the helicase WRN (Werner syndrome ATP-dependent RecQ like helicase) [120,121,122]. This activity is especially critical for the fork restart at telomeres [146]. Regulation of FEN1 activity is important in maintaining genome stability as overexpression of FEN1 is associated with poor prognosis in various cancers [137]. FEN1 overexpression results in impediment in replication fork progression, mid-S phase arrest, and hypersensitivity to DNA damaging agents [137].

### 5.2. Fan1 (*Schizosaccharomyces pombe*)/Absent in *Saccharomyces cerevisiae*/FAN1 (Fanconi-Associated Nuclease I) (Human)

FAN1 (Fanconi-associated nuclease I) is another structure-dependent endonuclease that plays a critical role in ICLs repair (reviewed in [148]) and in promoting replication fork progression in response to replication stress induced by agents such as HU and MMS [123,149]. There is no apparent FAN1 homolog in the budding yeast. FAN1 exhibits endonuclease activity toward 5ˇä flaps and has 5ˇä-3ˇä exonuclease activity [150]. A recent study suggests that FAN1 dimerizes to have optimal cleavage of a long 5ˇä flap strand [151]. FAN1 nuclease activity at stalled replication forks is tightly regulated as FAN1 activity is needed for fork restart but excessive activity can result in fork degradation (reviewed in [148]) [149]. *Fan1^−/−^* mice have repeat expansions in brain and other somatic tissues, demonstrating that FAN1 activity contributes to the maintenance of genome integrity [152]. Like SLX4, FAN1 has a UBZ motif which allows its association with monoubiquitylated FANCD2 and subsequent recruitment to at replication forks (Table 1) [123]. FAN1 can also be recruited to aphidicolin-stalled replication forks via FANCD2-BLM (Fanconi anemia group D2 protein- Bloom's helicase) complex independent of the UBZ domain [149]. FAN1 also contains PCNA interacting peptide (PIP) motif that allows its association with ubiquitylated PCNA accumulated at stalled replication forks [124].

### 5.3. Absent in *S. pombe*/Yen1/GEN1

Yen1*^S.c.^* (crossover junction endodeoxyribonuclease 1) in budding yeast and GEN1 (XPG-like endonuclease 1) in humans are SSEs that belong to the XPG/Rad2 family and define another Holliday junction resolvase that can process replication intermediates (reviewed in [153]). In MUS81-deficient human cells, GEN1 can induce DSB following replication stress which is opposed by the presence of SLX4 [142]. In budding yeast, Yen1*^S.c.^* is phosphorylated by Cdc28*^S.c.^* at G1/S transition which inactivates its nuclear localization signal (NLS), ensuring Yen1*^S.c.^* stays in the cytoplasm until anaphase (Table 1) [22,23,24]. Cdc14*^S.c.^* dephosphorylates Yen1*^S.c.^* at anaphase, allowing it to enter the nucleus [23]. In human cells, GEN1 contains a nuclear export signal (NES) and cannot access chromatin until the nuclear envelope is broken down during mitosis [25]. It is absent in fission yeast which may explain why meiosis is highly dependent on Mus81–Eme1 in fission yeast (reviewed in [26]).

It is important to remember that there may be nucleases that have not been previously implicated in replication stress that may also contribute to processing replication intermediates. For example, a recent study suggests that Artemis, a nuclease involved in non-homologous DNA end-joining (NHEJ) (reviewed in [154]), contributes to processing stalled DNA replication forks and prevent chromosomal segregation defect during mitosis [10]. Artemis is not present in yeast.

## 6. Concluding Remarks

**Figure 5:**
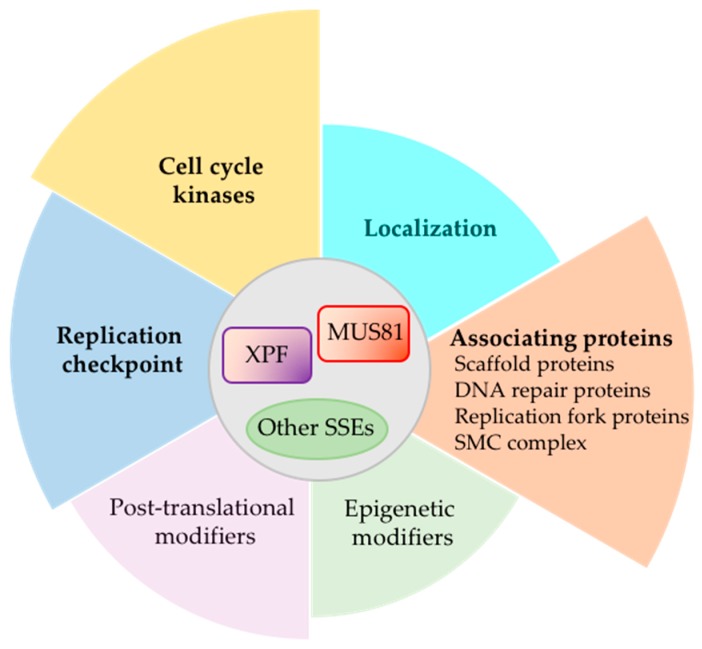
Summary of types of regulation of SSEs (MUS81 and XPF in particular) involved in resolving replication intermediates.

We have summarized findings showing how SSEs, MUS81 and XPF in particular, are controlled during cell cycle and replication stress (Figure 5). Cell cycle kinase, replication checkpoint kinase, and the various interacting partners as well as inducers of post-translational and epigenetic modifiers work in consortium, allowing cells to quickly respond to replication stress but limit extraneous DNA damage. Teasing out the regulatory networks that control SSE activities and how they communicate with each other can help gain a more comprehensive understanding of how SSEs contribute to cancer. On one hand, SSEs are needed to maintain genome stability but on the other hand, DNA cleavage by SSEs can contribute to inducing DNA damage and chromosome rearrangement. For example, Mus81 cleavage of the displacement loop (D-loop), the initial recombination intermediate that form in broken replication forks, limits mutagenic template switches that propels genome instability in cancers [155]. The ability of Mus81 to work with Rad27*^S.c.^* (FEN1 in human) and post-replication DNA repair protein, Rad18*^S.c.^*, to suppress repeat-mediated chromosomal rearrangements has been suggested to inhibit large inverted duplications of chromosomal segments observed frequently in cancers [156]. In other contexts, Mus81 activity can contribute to survivability of cancer cells. For instance, Mus81-mediated resolution of toxic intermediates resulting from break-induced replication in the absence of Srs2*^S.c.^* helicase increases cell viability [157].

There is somewhat conflicting evidence on how SSEs influence chemotherapy response. In various types of cancer cells, downregulation of XPF or MUS81 increases sensitivity to chemotherapeutic drugs cells via CHK1 pathway activation or stimulation of apoptosis [63,158,159]. However, there is also evidence that cytosolic DNA generated by MUS81 in prostate cancers stimulate immune response, potentially contributing to host rejection of cancer cells [160]. More in-depth understanding of how SSE activities are controlled will help formulate better predictions about their involvement in carcinogenesis and in patient-response to anti-cancer therapeutics.

Some of critical questions regarding SSEs still need to be addressed:

- How do regulation and roles of MUS81 and XPF differ between mitosis and meiosis?
- What molecular brakes exist that allow SSEs to process aberrant replication structures without deleterious DNA breakage?
- How does chromatin structure or components influence SSE recruitment and activity?
- How do SSEs coordinate or communicate with other SSEs and other DNA-remodeling enzymes?
- Do SSE activities contribute to checkpoint activation and cell cycle arrest? If so, what is the molecular mechanism?

Exploring these questions and other uncharacterized aspects of SSEs will garner exciting and important insights needed to integrate our understanding of the replication process, genome stability and the cell cycle.

## Figures and Tables

**Table 1:** Regulators of structure-specific endonucleases (SSEs) besides cell cycle kinases and Cds1*^S.p.^* homologs. There are many other regulators of Mus81 and other SSE activities besides cell cycle-dependent kinases. Proteins involved in DNA repair or replication are the most common regulators. (*S.c.: S. cerevisiae; S.p.: S. pombe*).

Grouping of Regulatory Protein or Stimulus	Protein or Stimulus that Regulates SSE Activities	Organism	Effect on SSE	Reference
Mus81-Eme1 (*S. p.*)/Mus81-Mms4 (*S. c.*)/MUS81-EME1 (human)				
Players of DNA repair or replication	Rad52	*S. c.,* Human	Stimulate activity	[94,95,96]
	Esc2	*S. c.*	Stimulate activity	[75]
	RFC/PCNA	*S. c.*	Stimulate activity	[97]
	FANCD2	Human	Promote recruitment & activity	Rev. in [98,99]
	RECQ5 helicase	Human	Promote recruitment to CFS	[65]
SMC protein complex	Smc5/6	*S. c.,* Human	Stimulate activity	[99,100]
	SMC2	Human	Promote recruitment	[101]
	WAPL	Human	Promote recruitment	[101]
	Psm1	*S. p.*	Stimulate activity	[102]
Localization	Nucleolar	Human	Maintains repetitive nucleolar DNA	[103]
	DNA damage-induced	*S. c.,* Human	Maintains genome stability after DNA damage	[103,104]
Post-translational modifier	SUMOylation	Human	Stimulate activity upon arsenic treatment	[105]
Epigenetic modifier	EZH2	Human	Methylation on H3K27 at stalled replication fork stimulate recruitment	[106]
Scaffold protein	SLX4	*S. c.,* Human	Promote recruitment & activity	[11,12,13,14,15,60]
	UHRF1	Human	Promote recruitment	[107,108]
Rad16-Swi10 (*S. p.*)/Rad1-Rad10 (*S. c.*)/XPF-ERCC1 (human)				
Players of DNA repair or replication	Rad14	*S. c.*	Promote recruitment	[109]
	RPA	Human	Stimulate activity	[110,111]
	Rad52	Human	Stimulate activity	[110,112]
	FANCD2	Human	Promote recruitment	Rev. in [98,99]
Scaffold protein	Pxd1	*S. p.*	Stimulate activity	[113]
	SLX4	*S. c.,* Human	Promote recruitment & activity	[11,12,13,14,15]
	UHRF1	Human	Promote recruitment	[107,108]
DNA binding protein	Saw1	*S. c.*	Promote recruitment	[114]
Rad2(*S. p.*)/Rad27(*S. c.*)/FEN1 (human)				
Players of DNA repair or replication	PCNA	Human	Promote recruitment & activity during Okazaki fragment maturation	Rev. in [115]
	Rad9-Rad1-Hus1 complex	Human	Promotes activity during replication stress	[116]
	MUS81	Human	Stimulate activity	[117,118,119]
	RECQ5 helicase WRN	Human	Promote recruitment & activity	[120,121,122]
Post-translational modifier	SUMOylation	Human	Promotes association with Rad9-Rad1-Hus1 complex	[116]
Fan1(*S. p.*)/Absent in *S. c.*/FAN1 (human)				
Players of DNA repair or replication	FANCD2	Human	Promote recruitment	[123]
	PCNA	Human	Promote recruitment	[124]
Absent in *S. p.*/Yen1 *S. c.*/GEN1 (human)				
Localization	Cdc28	*S. c.*	Nuclear exclusion at G1/S	[22,23,24]
	Cdc14	*S. c.*	Nuclear import at anaphase	[23]
	Nuclear Export Signal	Human	Nuclear exclusion until nuclear envelope breakdown	[25]
